# Increasing incidence and severity of childhood-onset type 1 diabetes in Latvia during the COVID-19 pandemic

**DOI:** 10.3389/fped.2026.1804669

**Published:** 2026-05-22

**Authors:** Lizete Braivo, Viktorija Truškova, Aļona Lavrenova, Anda Ķīvīte-Urtāne, Jana Pavāre, Iveta Dzīvīte-Krišāne

**Affiliations:** 1 Department of Paediatric Endocrinology, Children’s Clinical University Hospital, Riga, Latvia; 2 Department of Paediatrics, Rīga Stradiņš University, Riga, Latvia; 3 Department of Paediatric Infectious Diseases, Children’s Clinical University Hospital, Riga, Latvia; 4 Institute of Public Health, Rīga Stradiņš University, Riga, Latvia

**Keywords:** COVID-19 pandemic, diabetic ketoacidosis, pediatrics, SARS-CoV-2 virus, type 1 diabetes

## Abstract

**Background:**

During the COVID-19 pandemic in Latvia, there was a significant increase in new-onset childhood type 1 diabetes (T1D). The aim of this study is to analyze the influence of SARS-CoV-2 virus on initial manifestation of T1D and patterns of risk factors for disease development in children.

**Materials and methods:**

In the cross-sectional part of the study, 277 patients aged 1–17 years with new-onset T1D hospitalized in Children’s Clinical University Hospital between 2017 and 2024 were selected and divided into two groups depending on COVID-19 history prior to T1D onset: (1) research group (past, laboratory-proven infection or positive SARS-CoV-2 antibodies at the time of hospitalization); (2) control group (negative COVID-19 history and prepandemic patients). Initial manifestation was evaluated by analyzing clinical presentation and blood gas tests. Among the risk factors, prenatal, dietary, and hereditary factors and infections were analyzed.

**Results:**

The rate of incidence of T1D increased by 37.6% during the COVID-19 pandemic in Latvia (*p* < 0.001). Children from the research group had a significantly more severe initial manifestation with lower pH levels at the time of admission (*p* = 0.04). No association between more severe clinical presentation and delayed arrival at the hospital for any reason was found. There was a significantly lower prevalence of T1D in the family members of those patients exposed to SARS-CoV-2.

**Conclusion:**

After exposure to SARS-CoV-2, patients in the cohort group had a more severe initial manifestation of T1D than patients without a history of COVID-19, which could not be attributed to the restrictions imposed by the pandemic or to other changes resulting from patient or primary care decisions.

## 1 Introduction

Type 1 diabetes (T1D) is a chronic autoimmune disease with the highest incidence in the pediatric population. The overall rate of incidence is gradually increasing globally; in Europe, the increase is, on average, 2%–5% per year (1). However, since the outbreak of the COVID-19 pandemic, several countries and medical centers have reported an unexpectedly high increase in new-onset T1D cases. A systematic review by Daniel D'Souza et al. published in June 2023 reported a significantly higher incidence of T1D during the first year of the pandemic, compared with the prepandemic period, which had an incidence rate ratio of 1.14 (2). Other countries such as Germany, United Kingdom, and Ireland reported similar data (3, 4).

It is already proven that the development of T1D is influenced by both genetic and environmental factors such as infections and diet (5). SARS-CoV-2 virus was undoubtedly considered a possible reason for increasing cases of new-onset T1D. Several theories have been developed to explain this phenomenon. Initial studies about the influence of the SARS-CoV-2 virus on metabolism increasing the risk for hyperglycemia and both type 1 and type 2 diabetes were published as early as in 2020. The most common theory about the influence of COVID-19 on the development of diabetes pertains to the SARS-CoV-2 virus binding to the angiotensin-converting enzyme 2 receptor that is expressed in pancreatic *β* cells with higher cell damage (6). Another possible mechanism is the indirect SARS-CoV-2 virus effect causing cytokine storm with the consequent destruction of pancreatic cells (7). The SARS-CoV-2 virus may also suppress the excretion of insulin secretory granules (8) or stimulate the ongoing autoimmune process (3).

Indirect COVID-19 pandemic effects should also be mentioned as possible inducers for the development of T1D. An important factor was presumed to be increased psychological stressors due to social isolation and other restrictions (3). According to data from the World Health Organization (WHO), the global rate of prevalence of anxiety and depression increased by 25% during the pandemic (9). Similar data were reported in children and adolescents (10). Psychological stressors have already been implicated in the development of T1D (11). Another indirect effect of the pandemic was restricted contact with different environmental factors and infectious agents, leading to reduced biodiversity that was presumed to increase the risk for the development of immune-mediated diseases such as T1D (3).

A significant rise in new-onset T1D cases was observed in Latvia as well. As soon as the increase in incidence was noticed, our aim was to analyze whether the SARS-CoV-2 virus had influenced the initial manifestation of T1D and whether there had been changes in the spectrum of risk factors for the development of T1D, given the possibility of a new diabetogenic agent in the form of the SARS-CoV-2 virus.

## 2 Materials and methods

The dynamics of T1D incidence in Latvia were analyzed for the period between 2010 and 2024. Epidemiological changes that occurred during the active COVID-19 pandemic years from 2020 to 2022 were compared with those during the 10-year prepandemic period. The years 2023–2024 were excluded from this analysis as the number of new SARS-CoV2 cases was low, and in 5 May 2023, it was announced by the WHO that the COVID global health emergency had ended. Data about new-onset T1D cases in Latvia were obtained from the Children’s Clinical University Hospital (CCUH) database, as this is the only tertiary children hospital in Latvia where all new-onset type 1 diabetes patients are hospitalized and trained for disease control. Thus, these data reflect national incidence data from Latvia. The incidence of T1D was calculated per 100,000 person-years (py). Incidence rates across the two time periods were compared using the Compare Two Rates function in the OpenEpi open-access calculator, with statistical significance assessed using the Mid-*P* exact test (12).

The second part of this study was a closer evaluation of the influence of the COVID-19 pandemic on initial manifestation and risk factor spectrum for disease development. This was a cross-sectional study where patients 1–17 years of age with new-onset T1D were included retrospectively from 2017 and prospectively from August 2022 to January 2024. The patients were further divided into two groups: (1) the research group—patients with a positive COVID-19 history before manifestation of T1D (either patients with acute infection that was laboratory proven or those with positive SARS-CoV-2 virus-specific nucleocapsid antibodies by the time of the initial manifestation of T1D) and (2) the control group (those with a negative COVID-19 history and negative SARS-CoV-2 antibodies, as well as patients who had initial T1D manifestation before the COVID-19 pandemic).

**Figure 1:**
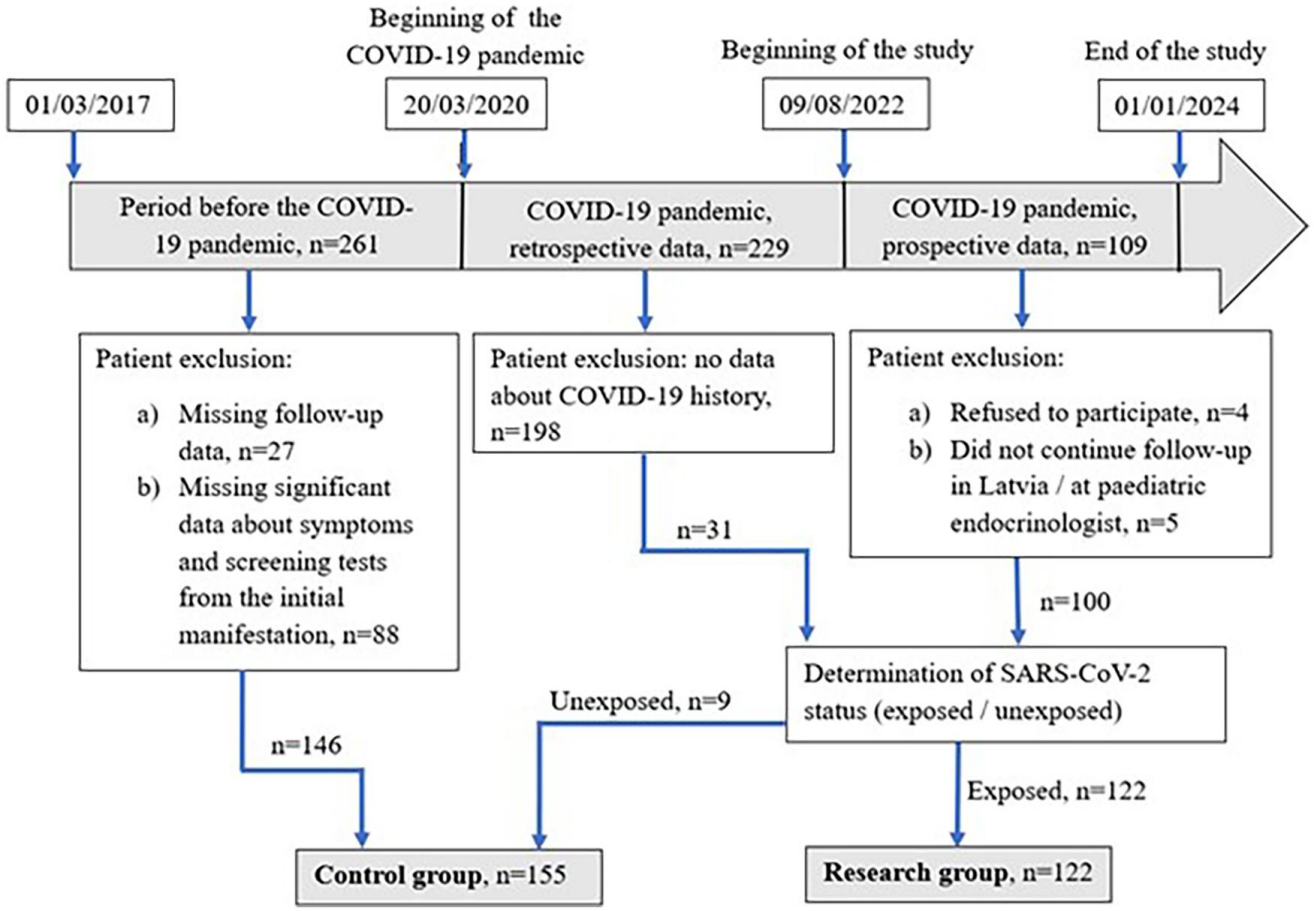
Patient enrolment in the study.

A retrospective patient enrollment became necessary to increase the numbers in the control group, because at the start of the study, nearly 90% of new-onset T1D patients had a positive COVID-19 history. Out of 599 patients who were newly diagnosed with T1D from March 2017 to January 2024 in Latvia, 277 were selected for further analysis. Patients were included prospectively only after they gave consent to participate in the study and if they were willing to being subjected to continued observations for 1 year, as this study continued with further evaluation of the disease course. A retrospective selection of patients within the pandemic period was made if the SARS-CoV-2 status was known, but patients belonging to the prepandemic period were selected for the control group if 1-year follow-up data were available for additional analysis. Data for retrospectively included patients were collected by using medical records, and in prospectively included patients, they were collected through interviews and medical records. Written informed consent was obtained from the parents of the patients. A patient enrollment is shown in Figure 1.

The following data were included, analyzed, and compared between the two groups: signs and symptoms before hospitalization, initial blood gas test results, initial full blood count, biochemistry analysis (including liver and kidney function test results, inflammatory markers, lipid profile, HbA1c test results), screening for autoimmune comorbidities (anti-TPO and anti-tTG IgA), and hormone levels [C-peptide, thyroid-stimulating hormone, 25(OH)D]. Dyslipidemia was defined according to ISPAD guidelines (13) if a patient experienced any of the following changes in their lipid profile: total cholesterol >5.17 mmol/L, triglycerides >1.7 mmol/L, low-density lipoproteins > 2.6 mmol/L, or high-density lipoproteins < 1.03 mmol/L. Severity of the initial manifestation was evaluated using capillary blood gas tests. Severe diabetic ketoacidosis (DKA) was defined according to the ISPAD guidelines (14). The education level of the patients’ parents was taken into account when analyzing the severity of the initial manifestation. To analyze possible changes in outpatient care caused by the COVID-19 pandemic, the role of the general practitioner (GP) in a prehospital setting was analyzed by conducting interviews with the following questions: (1) whether patients had sought help from the GP after the onset of symptoms, (2) availability of the GP, (3) diabetes recognition/GP's reaction to symptoms, and (4) reasons for delayed hospital presentation.

**Figure 2:**
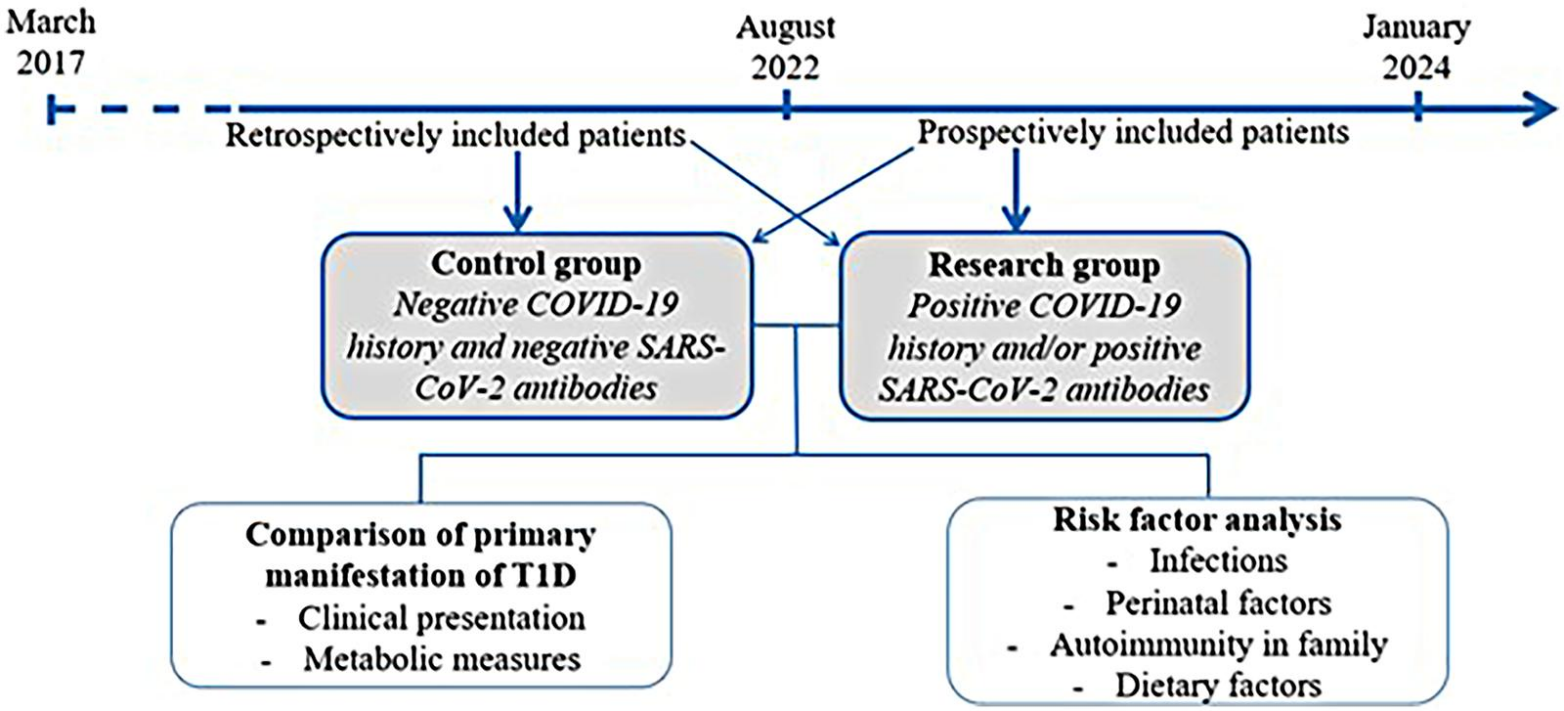
Patient selection and structure of the study.

The risk factors for T1D development, such as family history of T1D and other autoimmune diseases, perinatal history, dietary factors, and infections were analyzed and compared. Data were obtained from interviews and medical records. Specific information regarding perinatal history, including infants’ diet in the first year of life and autoimmune disease in family history, is routinely sought in the Department of Pediatric Endocrinology and documented in the medical records of patients. A family history of type 1 diabetes was accounted for in the first-and second-degree relatives of the patients and then specified. The patients were asked about the family history of other autoimmune diseases, including autoimmune thyroiditis, celiac disease, vitiligo, autoimmune adrenalitis, autoimmune arthritis, and others. In prospectively included patients, a more detailed history of infections 1 year before diagnosis was gathered, for example, frequency of infection episodes and type of infections (respiratory, gastrointestinal, enterovirus, and others). Data regarding SARS-CoV-2 infection before T1D onset were analyzed in the research group. For obtaining a more precise infection history, serology testing for already proven diabetogenic infections was performed: Epstein–Barr infection [EBV IgM (ZEBRA), EBV IgM (VCA), EBV IgG (VCA), EBV IgG (EBNA)], cytomegalovirus (CMV IgM, CMV IgG), Parvovirus B19 (IgM and IgG antibodies), Influenza A (IgA and IgG antibodies), Influenza B (IgA and IgG antibodies), Echovirus (IgA and IgM antibodies), and Coxsackie virus (IgA and IgM antibodies). The structure of this study is shown in Figure 2.

### 2.1 Sample size calculation

The sample size of the study was calculated using Kelsey and Fleiss formulas, based on previous publications on the incidence of diabetic ketoacidosis in pediatric patients during the COVID-19 pandemic and the prepandemic period (15). Because this is a part of a larger study that included additional observations about prospectively included patients, the sample size was calculated using cohort study calculations. With an alpha value of 0.05 and a beta value of 0.2, and assuming that the frequency of outcome (DKA) was 55% in the research group and 36.4% in the control group, the minimum sample size in each group was calculated to be between 113 and 123 (12, 16).

### 2.2 Statistical analysis

A statistical analysis was performed using Statistical Package for the Social Sciences (SPSS) version 23.0 (IBM SPSS Corp., Armonk, NY, USA). For parametric data, descriptive statistics with mean ± standard deviation (SD) was used. For non-parametric data, variables were expressed in median and interquartile range (IQR) (Q1–Q3). Data normality was assessed using the Kolmogorov–Smirnov test. To compare non-parametric data between the two groups, a Chi-Square test and Fisher’s exact test were performed. For the comparison of parametric data, the *t*-test was used. To compare continuous non-parametric data, the Mann–Whitney *U*-test was performed. To assess the correlation between parental level of education and severity of the initial manifestation, Spearman's correlation was used. A *p*-value of 0.05 was found to be statistically significant.

## 3 Results

**Figure 3:**
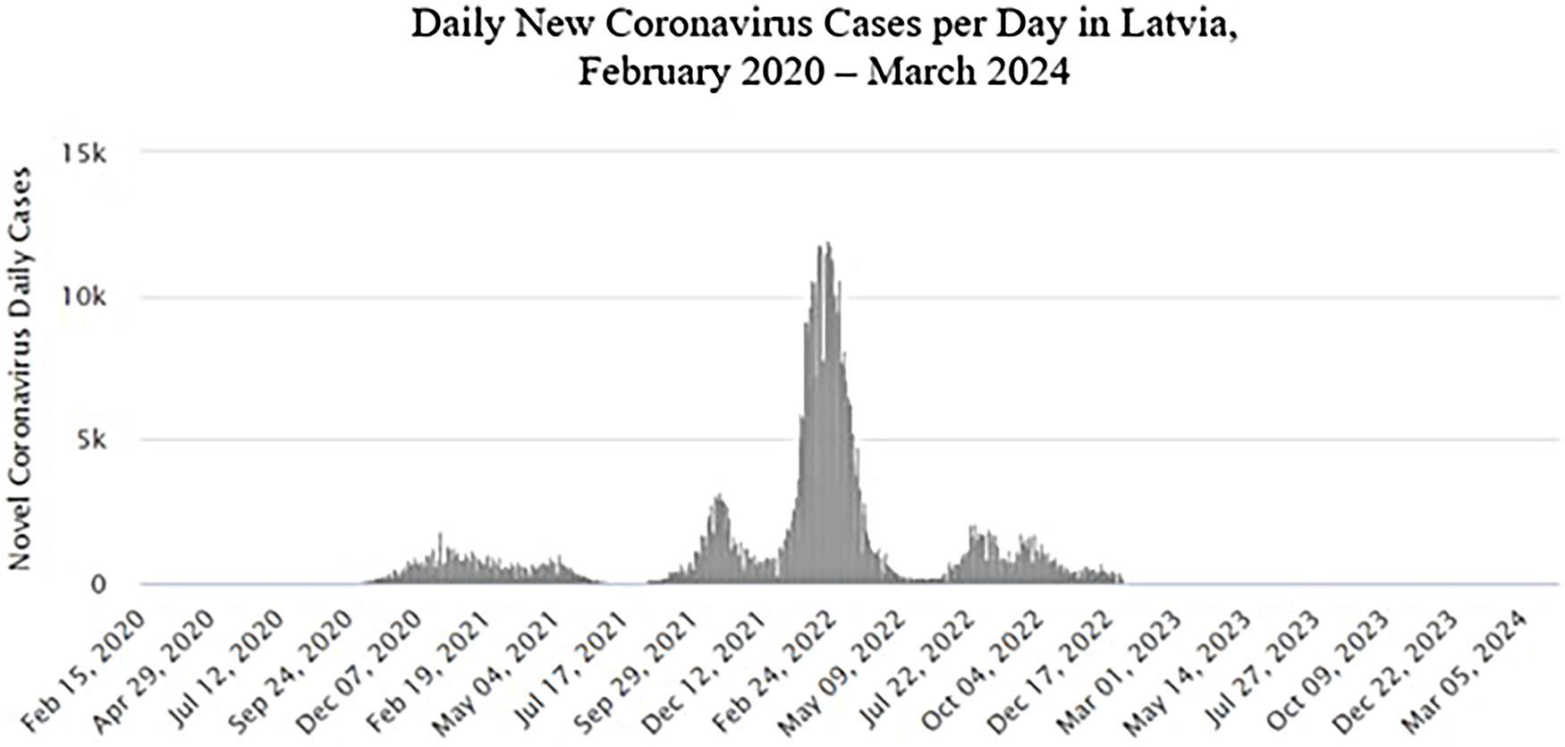
Daily New Coronavirus Cases per Day in Latvia from February 2020 to March 2024 (Worldometer) (17).

**Figure 4:**
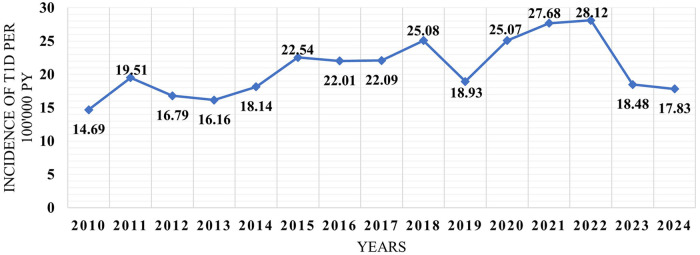
Incidence of type 1 diabetes in children, Latvia (2010–2024), per 100,000 person-years.

Data from the CCUH database showed an increase in the rate of T1D incidence by 37.62% since the outbreak of the COVID-19 pandemic, that is, from an incidence of 19.59 per 100,000 person-years during the 10-year-long prepandemic period to 26.96 since 2020 (*p* < 0.001). It is noteworthy that since the number of SARS-CoV-2 cases in Latvia dropped significantly in 2023 (less than 400 cases per week), the incidence of T1D also decreased to the prepandemic level. Data on SARS-CoV-2 registered active cases in Latvia are given in Figure 3 (17). The dynamics of the incidence of T1D in Latvia are reflected in Figure 4.

### 3.1 Study population

A total of 277 patients were included in this study: 122 (44%) in the research group (53.3% boys) and 155 (56%) in the control group (57.4% boys). Age differences were observed between the two groups, with a significantly smaller proportion of patients in the research group aged 1–4 and 10–14 years but a higher proportion of 5–9-year-olds in the control group. The significant difference in vaccination status against COVID-19 can be explained by the fact that most patients from the control group were included retrospectively from the prepandemic period. Patient characteristics at the time of initial manifestation are given in Table 1.

**Table 1:** Patient characteristics, demographic features, and comorbidities at the time of initial presentation .

Patient characteristic	Research group (*n* = 122)	Control group (*n* = 155)	Total (*n* = 277)	*P*-value
Age (years), mean (SD)	9.7 (4.5)	10.0 (4.6)	9.9 (4.6)	0.91
Age groups (%)				
1–4 years old	12.3	19.4	16.2	**0**.**04**
5–9 years old	36.1	21.3	27.8	
10–14 years old	35.2	42.6	39.4	
15–18 years old	16.4	16.8	16.6	
Sex—boys (%)	53.3	57.4	55.6	0.49
Body mass index in percentiles, median (IQR)	35 (8.0–68.0)	27 (4.0–65.0)	33.5 (5.0–67.0)	0.6
Chronic comorbidities	16.5	21.3	19.2	0.32
Cardiological (%)	1.6	0.6	1.1	0.58
Respiratory (%)	0.8	5.8	3.6	**0.04**
Celiac disease (%)	3.3	1.9	2.5	0.7
Autoimmune thyroiditis (%)	4.9	5.2	5.1	0.93
Vitiligo (%)	0	1.3	0.7	0.51
Vaccinated against COVID-19 (%)	16.7	1.3	20 (7.7)	**<0**.**001**

SD, standard deviation; IQR, interquartile range.

Bold values represent data with significant difference between the groups, *p* < 0.05.

### 3.2 Initial presentation of type 1 diabetes, differences depending on previous SARS-CoV-2 exposure, parents’ education level, and other factors

The median pH level in the blood gas test at the time of admission was significantly lower in patients with a history of COVD-19 (*p* = 0.045). The most remarkable differences were observed in patients aged 5–9 years (significantly lower pH levels in the research group) and those in the adolescent SARS-CoV-2-exposed group aged 15–18 years, with an overall higher percentage of patients presenting with moderate or severe DKA (35% vs. 4% in the control group, *p* = 0.03); patients of this age group were consequently more frequently admitted to the pediatric intensive care unit (PICU) (25% vs. 0% in the control group, *p* = 0.01). The duration of symptoms before hospitalization did not significantly differ between the groups. Multiple typical diabetes symptoms were significantly more prevalent in the research group, which may be linked to more severe initial manifestation. In adolescent patients with a previous COVID-19 history, behavioral and emotional changes were significantly more frequently observed. Clinical manifestation by the time of hospitalization is shown in Supplementary Table S1.

The level of parent's education was also evaluated as a possible influential factor for severity of DKA. A weak, but statistically significant, correlation was found between the mother's education level and severity of the initial manifestation: the higher the education level, the less severe the initial manifestation of T1D (*ρ* = −0.26, *p* = 0.008). A similar but moderate correlation was found between the father's education level and severity of DKA (*ρ*= −0.32, *p* = 0.002).

An analysis of the prehospital period showed that 40 patients (43.5%) from the research group did not seek care from the GP for their diabetes symptoms. This group was not associated with a higher prevalence of DKA at the time of hospitalization (*p* = 0.21). However, we do not associate this phenomenon with the restrictions imposed by the COVD-19 pandemic as our analysis shows that during the pandemic period, families sought help from the GP even more frequently than during the non-pandemic period (55% vs. 39.7% accordingly). We also analyzed the decisions made by the GPs to whom children with diabetes symptoms turned initially. Most GPs (70.7%) decided to perform laboratory tests (blood or urine) or referred patients directly to the hospital (25.9%). In two cases (both from the research group), diabetes symptoms were not recognized and therefore no further recommendations were given. In eight cases (seven from the research group) in which children were referred to undergo laboratory tests, pathological changes in the results were not noticed by the GP, and these patients were not immediately referred to the hospital. Four (50%) of these patients initially presented with DKA. An analysis of those with delayed arrival at the hospital (either primary care associated or patient associated—afraid of long waiting queues, not aware of the seriousness of the situation) did not show any association with more severe initial presentation. The prevalence of delayed presentation at the hospital for any reason was similar between the SARS-CoV-2-exposed and the unexposed groups. The analysis did not find any other significant factor that could explain the more severe initial manifestation of T1D in the SARS-CoV-2-exposed children.

An initial metabolic screening showed a significantly lower C-peptide level in the research group of patients aged 1–4 years and a noticeably higher prevalence of dyslipidemia in those aged 15–18 who had been previously exposed to SARS-CoV-2. A comparison of other laboratory findings between the research groups can be found in Supplementary Table S2. Basic biochemistry tests did not show any significant differences between the two groups, except with regard to the albumin level, which was significantly lower in the research group at the time of initial manifestation.

### 3.3 Risk factor analysis

A T1D risk factor evaluation was performed in order to equalize the research and control groups and to rule out the significant effects of potential risk factors other than COVID-19 for T1D development. The evaluation included an analysis of perinatal factors, dietary factors, infections, and family history of T1D playing a possible genetic role in T1D development.

**Figure 5:**
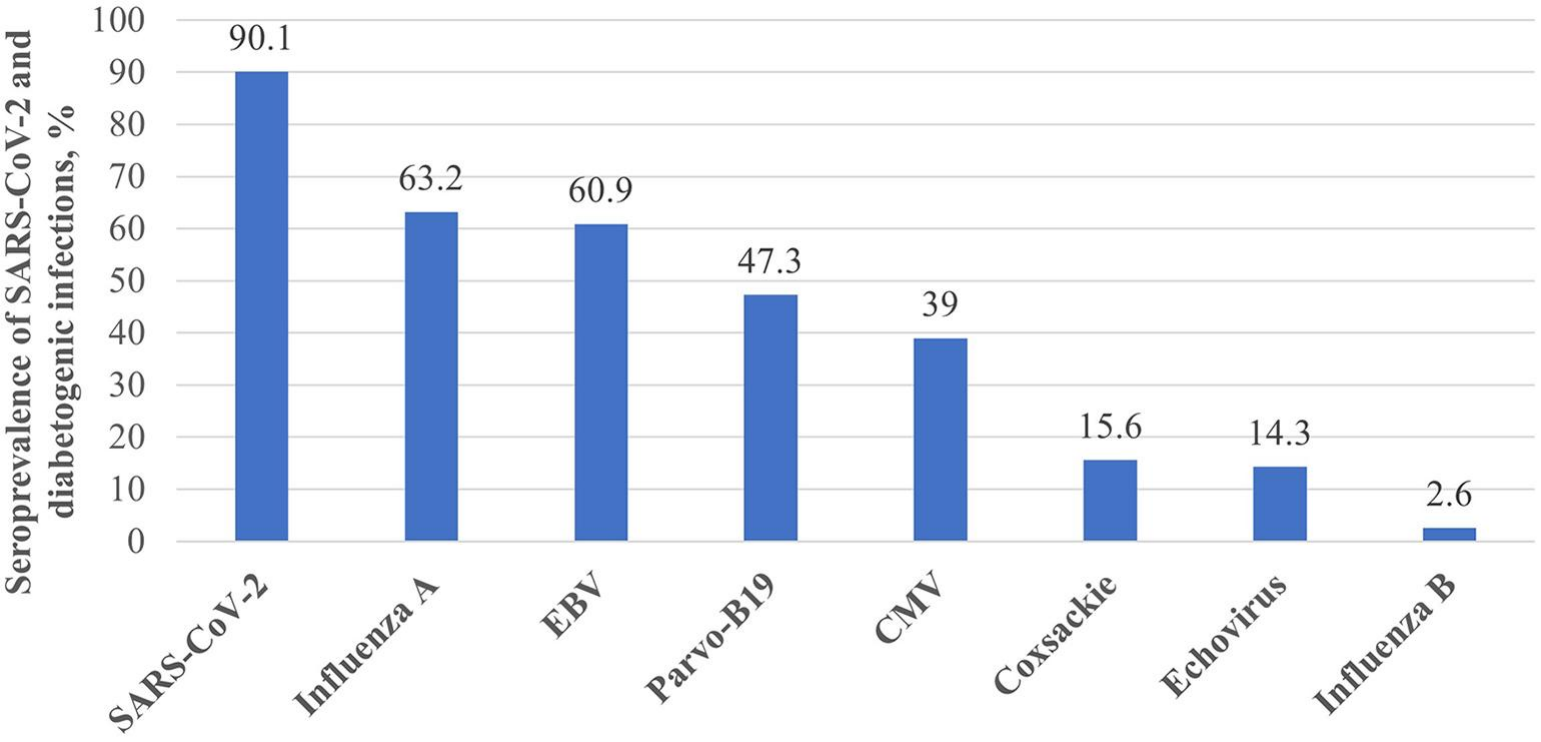
Seroprevalence of diabetogenic infections in prospectively included patients.

Children with previous SARS-CoV-2 exposure had a positive family history of T1D significantly less frequently (8.2% vs 16.8% in controls, *p* = 0.04), which may reveal a lesser extent of genetic involvement in the development of T1D in these patients. The prevalence of other autoimmune diseases among first- and second-degree family members was similar between the groups. Among the perinatal risk factors, jaundice was observed significantly more often in the research group. However, such differences may be explained by uneven data collection, because in the research group, most patients were purposefully asked about their perinatal history, but in the control group, most data were collected through medical documentation and presumably were not mentioned. Among the dietary factors, early introduction of cow milk and the level of 25(OH)D were analyzed, and the analysis did not show significant differences between the groups. Serology testing showed that 90.1% of all prospectively included patients were seropositive for SARS-CoV-2. The median time from COVID-19 and the first manifestation of T1D was 33.0 (15.0–69.0) weeks. Patients from the research group had a significantly longer duration between the preceding infection and the first symptoms of T1D than those from the control group. The seroprevalence of infections is shown in Figure 5. A comparison of T1D risk factors is presented in Table 2.

**Table 2:** Comparison of type 1 diabetes risk factors between the research and control groups.

Risk factor	Research group (*n* = 122)	Control group (*n* = 155)	Total (*n* = 277)	*P*-value
Perinatal risk factors				
Mother age >25 years at the time of patients’ birth (%)	70.1	70.3	70.2	0.97
Preeclampsia (%)	1.8	2.7	2.1	0.99
Neonatal respiratory distress (%)	2.7	1.4	2.2	0.65
Neonatal jaundice (%)	16.2	5.4	11.9	**0**.**03**
Maternal gestational diabetes (%)	6.6	1.3	4.6	0.16
Dietary factors				
Early introduction of cow milk (<3 months of age) (%)	27.3	38.9	31.9	0.1
Early introduction of cow milk (<6 months of age) (%)	40.0	52.1	44.8	0.11
Level of 25(OH)D, ng/mL, median (IQR)	22.4 (16.0–31.9)	23.3 (14.5–31.1)	22.5 (15.1–31.2)	0.61
Family history of autoimmune diseases				
Autoimmune diseases in family (first- and second-degree relatives) (%)	27.9	27.1	27.4	0.89
Type 1 diabetes in family (first- and second-degree relatives) (%)	8.2	16.8	13.0	**0**.**04**
Infections during the 1-year period before T1D manifestation				
History of any infection during the 1-year period before T1D manifestation (%)	88.7	95.7	90.7	0.24
Respiratory tract infection (%)	73.3	94.4	78.8	**0**.**008**
Gastroenteritis (%)	13.4	47.6	19.5	**0**.**001**
Enterovirus infection (%)	1	7.1	1.8	0.24
Time between last infection and first manifestation of T1D, weeks, median (IQR)	12.0 (4.0–22.0)	3.5 (1.0–8.0)	8.0 (3.0–16.5)	**<0**.**001**

IQR, interquartile range; T1D, type 1 diabetes mellitus.

Bold values represent data with significant difference between the groups, *p* < 0.05.

## 4 Discussion

This cross-sectional study analyzed potential SARS-CoV-2 influence on the initial manifestation and development of T1D in children. In comparison with other similar studies that divided patients into prepandemic and pandemic groups and compared their initial manifestation, our classification was based on known COVID-19 history or SARS-CoV-2 seropositivity in patients with new-onset T1D.

Epidemiology studies showed a significant increase in the incidence of T1D in children during the COVID-19 pandemic by 37.6%, compared with the 10-year prepandemic period. Similar data have been presented in several other studies. A systematic review and meta-analysis published in June 2023 by Kamrath et al. presented data from 17 studies including 38,149 children with new-onset T1D and found a 16% (95% CI, 10%–23%) higher incidence rate of childhood T1D during the first 12 months and a 28% (95% CI, 18%–39%) higher incidence rate during the subsequent 12 months of the pandemic in comparison with the year before the COVID-19 pandemic (18).

It is noteworthy that patients aged 10 years and older with previous SARS-CoV-2 exposure experienced significantly higher behavioral changes and mood swings before a diagnosis of T1D was made. Glucose fluctuations may explain such symptoms, but another possible reason for these could be the restrictions imposed by the pandemic and social isolation and other factors that may influence children's mental health and presumably the development of T1D as well. Several researchers have already described the influence of psychological stressors on the development of T1D (19, 20).

Severity of the initial manifestation that was mainly evaluated based on blood gas test results that revealed that patients with previous SARS-CoV-2 exposure had more severe initial manifestation, especially those aged 5–9 years and 15 years and older. Increasing severity of the initial manifestation during the COVID-19 pandemic has been recognized in multiple studies. Research in the United Kingdom and Ireland conducted by Ponmani et al. that was published in May 2023 concluded that there was an increase in the incidence of DKA in children presenting with new-onset diabetes by 43% and in severe DKA by 79%, as well as increased admissions to the PICU by 89% (4). Another meta-analysis published by D'Souza et al. reported an increase in the incidence of DKA during the COVID-19 pandemic by 26% (2). Similar to the data published by Ponmani et al., we did not find any association between severity of the initial manifestation of T1D and delayed presentation at the hospital for any reason (4).

A risk factor analysis revealed that patients with previous SARS-CoV-2 exposure had a positive family history of T1D significantly less frequently. This may reveal a reduced genetic role in the development of T1D in patients from the research group. Further studies should undoubtedly be performed in order to analyze the genetic predisposition in these patients. Serostudies showed that SARS-CoV-2 had the highest rate of antibodies in prospectively included patients. This, of course, does not explain the development of T1D. However, COVID-19 was one of the most common infections among children during the pandemic. In contrast, the incidence of other infections during the pandemic was significantly lower than usual. An analysis of the infections that preceded T1D symptoms showed that patients from the research group had a significantly longer duration between infection and clinical diabetes (12 vs. 3.5 weeks in the control group, respectively), and many of them (at least 28.7%) had SARS-CoV-2 as a preceding infection. A study from China published by Yang et al. analyzed infectious diseases during the 407-day period before the onset of type 1 diabetes and concluded that there is a transient, but significantly increased risk of type 1 diabetes during the 42-day period after the infection, suggesting the role of infections as a precipitating factor for the ongoing autoimmune process (21). This may suggest that SARS-CoV-2 could have different pathophysiological effects on the development of T1D. Because it is already proven that infections may trigger progression of the autoimmune process, we cannot exclude the possibility that SARS-CoV-2 has a diabetogenic characteristic.

It is noteworthy that since the number of SARS-CoV-2 cases in Latvia dropped significantly in 2023, the incidence of T1D also decreased to the prepandemic level. Because we suspected SARS-CoV-2 to be a new diabetogenic microorganism, the decrease in T1D numbers may be explained by the fact that most children would have already been exposed to SARS-CoV-2 and virus-induced immunogenic effects are recognizable. Another explanation is that we saw a surge of patients with new-onset T1D during the pandemic who were previously predisposed to disease development, but different risk factors may have influenced a more rapid development of T1D.

The limitation of our study design was that at the start of this research, approximately 80%–90% of patients had already been exposed to SARS-CoV-2. As a result, most patients in the control group were included from the prepandemic period and data about their initial manifestation and risk factors were collected from medical documentation. The database is evenly distributed between the research and control groups, but for realizing our aim of a more accurate clinical presentation and risk factor evaluation, we ensured that prospectively included patients answered more specific questions that are not routinely asked and recorded in medical documentation. This may explain some of the differences in the clinical presentation and symptom prevalence between the two groups.

## 5 Conclusion

The incidence of childhood T1D in Latvia during the COVID-19 pandemic was significantly higher than in the prepandemic period. Children after SARS-CoV-2 exposure had a significantly more severe initial manifestation of T1D, and these changes in presentation were not associated with the restrictions caused by the pandemic or other reasons for a delayed referral to hospital. Patients previously exposed to SARS-CoV-2 had a significantly less common family history of T1D that may represent a lesser influence of genetic factors on the development of T1D in this group.

## Data Availability

The raw data supporting the conclusions of this article will be made available by the authors, without undue reservation.

## References

[1] Type 1 diabetes mellitus in children and adolescents: Epidemiology, presentation, and diagnosis. (2025). Available online at: https://www-uptodate-com.db.rsu.lv/contents/type-1-diabetes-mellitus-in-children-and-adolescents-epidemiology-presentation-and-diagnosis?search=type+1+diabetes+children&usage_type=default&source=search_result&selectedTitle=1%7E150&display_rank=1 (accessed February 4, 2026).

[2] D’Souza D Empringham J Pechlivanoglou P Uleryk EM Cohen E Shulman R. Incidence of diabetes in children and adolsecents during COVID-19 pandemic: a systematic review and meta-analysis. JAMA Netw Open. (2023) 6(6):e2321281. 10.1001/jamanetworkopen.2023.21281 37389869 PMC10314307

[3] Kamrath C Rosenbauer J Eckert AJ Siedler K Bartelt H Klose D Incidence of type 1 diabetes in children and adolescents during the COVID-19 pandemic in Germany: results from the DPV registry. Diabetes Care. (2022) 45(8):1762–71. 10.2337/dc21-0969 35043145

[4] Ponmani C Nijman EG Roland D Barett M Hulse T Whittle V. Children presenting with diabetes and diabetic ketoacidosis to emergency departments during the COVID-19 pandemic in the UK and Ireland: an international retrospective observational study. Arch Dis Child. (2023) 108(10):799–807. 10.1136/archdischild-2022-325280 37197894

[5] Type 1 diabetes mellitus: pathophysiology and etiology. UpToDate. (2026). Available online at: https://www-uptodate-com.db.rsu.lv/contents/type-1-diabetes-mellitus-pathophysiology-and-etiology (Accessed January 15, 2026).

[6] Liao Y-H Zheng J-Q Zheng C-M Lu K-C Chao Y-C. Novel molecular evidence related to COVID-19 in patients with diabetes Mellitus. J Clin Med. (2020) 9(12):3962. 10.3390/jcm9123962 33297431 PMC7762278

[7] Prosperi S Chiarelli F. COVID-19 and diabetes in children. Ann Pediatr Endocrinol Metab. (2022) 27(3):157–68. 10.6065/apem.2244150.075 36203266 PMC9537670

[8] Müller JA Groß R Conzelmann C Krüger J Merle U Steinhart J SARS-CoV-2 infects and replicates in cells of the human endocrine and exocrine pancreas. Nat Metab. (2021) 3:149–65. 10.1038/s42255-021-00347-1 33536639

[9] COVID-19 pandemic triffers 25% increase in prevalence of anxiety and depression worldwide (2022). Available online at: https://www.who.int/news/item/02-03-2022-covid-19-pandemic-triggers-25-increase-in-prevalence-of-anxiety-and-depression-worldwide (Accessed January 15, 2026).

[10] Racine N McArthur BA Cooke JE Eirich R Zhu J Madigan S. Global prevalence of depressive and anxiety symptoms in children and adolescents during COVID-19. JAMA Pediatr. (2021) 175(11):1142–50. 10.1001/jamapediatrics.2021.2482 34369987 PMC8353576

[11] Sharif K Watad A Coplan L Amital H Shoenfeld Y Afek A. Psychological stress and type 1 diabetes mellitus: what is the link? Expert Rev Clin Immunol. (2018) 14(12):1081–8. 10.1080/1744666X.2018.1538787 30336709

[12] Dean AG Sullivan KM Soe MM. OpenEpi: Open Source Epidemiological Statistics for Public Health (2013) Available online at: www.OpenEpi.com (Accessed November 5, 2025).

[13] Bjornstad P Dart A Donaghue KC Dost A Feldman EL Tan GS ISPAD Clinical practice consensus guidelines 2022: microvascular and macrovascular complications in children and adolescents with diabetes. Pediatr Diabetes. (2022) 23(8):1432–50. 10.1111/pedi.13444 36537531

[14] Glaser N Fritsch M Priyambada L Rewers A Cherubini V Estrada S ISPAD Clinical practice consensus guidelines 2022: diabetic ketoacidosis and hyperglycemic hyperosmolar state. Pediatr Diabetes. (2022) 23(7):835–56. 10.1111/pedi.13406 36250645

[15] Mastromauro C Blasetti A Primavera M Ceglie L Mohn A Chiarelli F Peculiar characteristics of new-onset type 1 diabetes during COVID-19 pandemic. Ital J Pediatr. (2022) 48(1):26. 10.1186/s13052-022-01223-8 35139895 PMC8827260

[16] Kelsey JL Whittemore AS Evans AS Thompson WD. Methods in Observational Epidemiology. 2nd edn. New York: Oxford University Press (1996).

[17] Worldometer. Coronavirus cases. Daily New Cases in Latvia. Available online at: https://www.worldometers.info/coronavirus/country/latvia/#graph-cases-daily (Accessed November 12, 2025).

[18] Kamrath C Holl RW Rosenbauer J. Elucidating the underlying mechanisms of the marked increase in childhood type 1 diabetes during the COVID-19 pandemic-the diabetes pandemic. JAMA Netw Open. (2023) 6(6):e2321231. 10.1001/jamanetworkopen.2023.21231 37389881

[19] Nygren M Carstensen J Koch F Ludvigsson J Frostell A. Experience of a serious life event increases the risk for childhood type 1 diabetes: the ABIS population-based prospective cohort study. Diabetologia. (2015) 58:1188–97. 10.1007/s00125-015-3555-2 25870022

[20] Sipetic S Vlajinac H Marinkovi J Kocev N Milan B Ratkov I Stressful life events and psychological dysfunctions before the onset of type 1 diabetes mellitus. J Pediatr Endocrinol Metab. (2007) 20(4):527–34. 10.1515/JPEM.2007.20.4.527 17550217

[21] Yang Z Zhou F Dorman J Wang H Zu X Mazumdar S Association between infectious diseases and type 1 diabetes: a case-crossover study. Pediatr Diabetes. (2006) 7(3):146–52. 10.1111/j.1399-543X.2006.00163.x 16787521 PMC7167653

