# Supplementary material for: Increasing incidence and severity of childhood-onset type 1 diabetes in Latvia during the COVID-19 pandemic
Source: Front Pediatr. 2026 May 22;14:1804669. doi: 10.3389/fped.2026.1804669 (PMC13236654; doi:10.3389/fped.2026.1804669)
Supplement: Supplementary file 1 [file Table1.docx]

Supplementary Material

## Supplementary Table 1. Clinical presentation of type 1 diabetes in children, comparison between research groups (R – research group, previously exposed to SARS-CoV-2; C – control group), divided by age periods (y.o. – years old; Total – all age groups included) (PICU = paediatric intensive care unit; DKA = diabetic ketoacidosis)

| **Signs and symptoms** | **1-4 y.o.** | | **P value** | **5-9 y.o.** | | **P value** | **10-14 y.o.** | | **P value** | **15-18 y.o.** | | **P value** | **Total** | | **P value** |
| --- | --- | --- | --- | --- | --- | --- | --- | --- | --- | --- | --- | --- | --- | --- | --- |
|  | **R**  (n=15) | **C**  (n=30) |  | **R**  (n=44) | **C**  (n=33) |  | **R**  (n=43) | **C**  (n=66) |  | **R**  (n=20) | **C**  (n=26) |  | **R**  (n=122) | **C**  (n=155) |  |
| Duration of symptoms, days, median (IQR) | 14 (5-21) | 14 (7-20.3) | 0.88 | 14 (7-30) | 14 (7-21.8) | 0.45 | 20 (10-42) | 14.5 (9.3-30) | 0.64 | 30 (14-56.3) | 30 (15-70) | 0.60 | 19  (7-30) | 14  (7-30) | 0.6 |
| Polydipsia, % | 93.3 | 93.3 | 0.99 | 88.6 | 84.8 | 0.74 | 88.4 | 86.4 | 0.76 | 90 | 76 | 0.27 | 89.3 | 85.7 | 0.37 |
| Polyuria, % | 93.3 | 83.3 | 0.65 | 86.4 | 78.8 | 0.38 | 83.7 | 72.7 | 0.18 | 90 | 69.2 | 0.15 | 86.9 | 75.5 | **0.02** |
| Polyphagia, % | 33.3 | 23.3 | 0.5 | 47.7 | 12.1 | **<0.001** | 41.9 | 15.2 | **0.002** | 25.0 | 24.0 | 0.99 | 40.2 | 17.5 | **<0.001** |
| Nocturia, % | 80.0 | 50.0 | 0.053 | 84.1 | 57.6 | **0.01** | 81.4 | 59.1 | **0.02** | 90.0 | 57.7 | **0.02** | 83.6 | 56.8 | **<0.001** |
| Enuresis, % | 40.0 | 10.0 | **0.04** | 47.7 | 36.4 | 0.32 | 11.6 | 7.6 | 0.51 | 0 | 0 | - | 26.2 | 13 | **0.01** |
| Prevalence of weight loss, % | 60.0 | 50.0 | 0.53 | 68.2 | 66.7 | 0.89 | 79.1 | 68.2 | 0.21 | 90.0 | 69.2 | 0.15 | 74.6 | 64.5 | 0.07 |
| Weight loss in % from initial weight (median, IQR) | 4.3 (0-7.5) | 0 (0-8.9) | 0.86 | 5.3 (0-14.1) | 3.2 (0-10.0) | 0.4 | 8.2 (2.1-15.2) | 8.0 (0-11.8) | 0.41 | 8.9 (7.2-15.4) | 7.1 (0-13.7) | 0.08 | 8.0 (0-14.6) | 6.3 (0-11.1) | 0.07 |
| Fatigue, % | 80.0 | 73.3 | 0.73 | 77.3 | 66.7 | 0.3 | 74.4 | 72.7 | 0.85 | 80.0 | 61.5 | 0.18 | 77 | 69.7 | 0.17 |
| Abdominal pain, % | 33.3 | 13.3 | 0.14 | 47.7 | 18.2 | **0.007** | 44.2 | 34.8 | 0.33 | 20.0 | 19.2 | 0.99 | 40.2 | 24.5 | **0.01** |
| Vomiting, % | 26.7 | 36.7 | 0.5 | 25.0 | 18.2 | 0.48 | 34.9 | 28.8 | 0.5 | 20.0 | 11.5 | 0.68 | 27.9 | 25.2 | 0.61 |
| Anorexia, % | 40.0 | 30.0 | 0.5 | 27.3 | 12.1 | 0.11 | 34.9 | 19.7 | 0.08 | 50.0 | 15.4 | **0.01** | 35.2 | 19.4 | **0.003** |
| Vision disturbances, % | 6.7 | 0 | 0.33 | 0 | 3.0 | 0.43 | 7.0 | 1.5 | 0.3 | 30.0 | 0 | **0.004** | 8.3 | 1.3 | **0.01** |
| Headache, % | 6.7 | 0 | 0.33 | 4.5 | 9.1 | 0.65 | 16.3 | 12.1 | 0.54 | 10.0 | 19.2 | 0.45 | 9.8 | 10.3 | 0.89 |
| Dizziness, % | 0 | 0 | - | 2.3 | 3.0 | 0.99 | 7.0 | 7.6 | 0.99 | 5.0 | 15.4 | 0.37 | 4.1 | 6.5 | 0.39 |
| Behavioural disturbances, mood swings, % | 46.7 | 23.3 | 0.17 | 38.6 | 18.2 | 0.052 | 46.5 | 21.2 | **0.005** | 60.0 | 3.8 | **<0.001** | 45.9 | 18.1 | **<0.001** |
| Pain and/or muscle spasms in legs, % | 0 | 3.3 | 0.99 | 2.3 | 0 | 0.99 | 0 | 4.5 | 0.28 | 10.0 | 15.4 | 0.68 | 1.9 | 5.2 | 0.36 |
| Infection, % | 6.7 | 33.3 | 0.07 | 9.1 | 3.0 | 0.39 | 7.0 | 9.1 | 0.99 | 15.0 | 11.5 | 0.99 | 9.0 | 12.9 | 0.31 |
| Kussmaul breathing, % | 33.3 | 30.0 | 0.99 | 34.1 | 21.2 | 0.22 | 28.6 | 22.7 | 0.49 | 30.0 | 0 | **0.004** | 31.4 | 20 | **0.03** |
| Lethargy, % | 46.7 | 43.3 | 0.83 | 36.4 | 27.3 | 0.4 | 27.9 | 36.4 | 0.36 | 40.0 | 3.8 | **0.006** | 35.2 | 30.3 | 0.39 |
| Shock, % | 26.7 | 23.3 | 0.99 | 20.5 | 12.1 | 0.33 | 19 | 24.2 | 0.53 | 25.0 | 0 | **0.01** | 21.5 | 17.4 | 0.4 |
| Admitted to PICU, % | 60.0 | 43.3 | 0.29 | 29.5 | 21.2 | 0.41 | 30.2 | 28.8 | 0.87 | 25.0 | 0 | **0.01** | 32.8 | 25.2 | 0.16 |
| **Severity of DKA** | | | | | | | | | | | | | | | |
| No DKA, % | 33.3 | 50.0 | 0.56 | 40.9 | 63.6 | 0.12 | 55.8 | 46.2 | 0.36 | 45.0 | 76.0 | **0.03** | 45.9 | 55.6 | 0.3 |
| Mild DKA, % | 26.7 | 16.7 |  | 13.6 | 15.2 |  | 16.3 | 18.5 |  | 20.0 | 20.0 |  | 17.2 | 17.6 |  |
| Moderate DKA, % | 33.3 | 20.0 |  | 22.7 | 6.1 |  | 7.0 | 18.5 |  | 15.0 | 4.0 |  | 17.2 | 13.7 |  |
| Severe DKA, % | 6.7 | 13.3 |  | 22.7 | 15.2 |  | 20.9 | 16.9 |  | 20.0 | 0 |  | 19.7 | 13.1 |  |
| **Initial capillary blood gas test** | | | | | | | | | | | | | | | |
| pH (median, IQR) | 7.29 (7.15-7.37) | 7.32 (7.17-7.39) | 0.62 | 7.27 (7.15-7.38) | 7.35 (7.23-7.41) | **0.03** | 7.33 (7.21-7.37) | 7.32 (7.17-7.39) | 0.78 | 7.26 (7.13-7.38) | 7.36 (7.31-7.40) | **0.02** | 7.29 (7.16-7.38) | 7.34 (7.19-7.4) | **0.045** |
| Glucose, mmol/l (median, IQR) | 21.0 (11.7-28.1) | 22.2 (15.0-29.7) | 0.71 | 23.6 (17.1-31.6) | 17.3 (12.7-25.8) | **0.01** | 19.0 (13.2-27.1) | 18.5 (14.9-23.9) | 0.85 | 23.1 (17.1-26.7) | 17.4 (13.1-22.6) | **0.02** | 22.1 (14.9-28.4) | 18.4 (14.6-25.0) | **0.02** |
| HCO3-, mmol/l (median, IQR) | 14.9 (9.4-19.4) | 16.4 (9.7-19.9) | 0.66 | 14.6 (10.0-20.6) | 18.5 (11.6-25.8) | 0.053 | 17.3 (11.5-21.3) | 15.2 (10.1-22.2) | 0.89 | 14.1 (11.2-22.0) | 20.1 (15.0-22.6) | 0.07 | 15.2 (10.8-21.0) | 17.7 (10.8-22.2) | 0.1 |
| BE, mmol/l (median, IQR) | -14.4 (-24.1-(-6.9)) | -12.2 (-22.3-(-6.9)) | 0.74 | -14.6 (-22.9-(-5.5)) | -8.9 (-20.0-(-2.6)) | 0.051 | -10.4 (-20.0-(-4.6)) | -14.5 (-22.9-(-3.3)) | 0.83 | -15.3 (-21.7-(-3.6)) | -6.3 (-14.3-(-2.2)) | 0.09 | -13.7 (-21.8-(-5.0)) | -9.8 (-21.0-(-3.3)) | 0.11 |
| Anion gap, mmol/l (mean, SD) | 24.0 (4.0) | 27.3 (5.3) | 0.09 | 25.1 (6.1) | 24.6 (5.6) | 0.81 | 24.7 (5.9) | 28.6 (5.6) | 0.73 | 26.7 (6.7) | 24.5 (5.5) | 0.4 | 25.1 (5.9) | 26.9 (5.8) | 0.9 |
| **Initial metabolic measures** | | | | | | | | | | | | | | | |
| HbA1c, % (mean, SD) | 10.7 (1.0) | 10.4 (1.5) | 0.27 | 12.2 (2.7) | 12.3 (2.5) | 0.52 | 13.7 (2.4) | 13.6 (3.9) | 0.12 | 13.6 (2.8) | 14.0 (4.3) | 0.053 | 12.8 (2.6) | 12.8 (3.6) | 0.051 |
| C-peptide, mean (SD) | 0.2 (0.1) | 0.3 (0.2) | **0.02** | 0.3 (0.2) | 0.3 (0.2) | 0.68 | 0.4 (0.2) | 0.4 (0.2) | 0.82 | 0.5 (0.4) | 0.6 (0.5) | 0.21 | 0.3 (0.2) | 0.4 (0.3) | 0.34 |
| Dyslipidaemia, % | 44.4 | 20.0 | 0.36 | 55.6 | 44.0 | 0.38 | 51.3 | 32.4 | 0.1 | 82.4 | 45.5 | 0.02 | 47.5 | 23.4 | **<0.001** |
| Hypophosphatemia, % | 13.3 | 8.7 | 0.99 | 21.1 | 21.1 | 0.99 | 25.0 | 26.2 | 0.9 | 31.6 | 6.7 | 0.1 | 23.2 | 18.2 | 0.37 |

**Supplementary Table 2.** Comparison of laboratory findings between SARS-CoV-2 exposed and non-exposed new-onset type 1 diabetes patients

| **Laboratory test** | **Research group** (n=122) | **Control group** (n=155) | **Total** (n=277) | **P-value** |
| --- | --- | --- | --- | --- |
| Leukocyte count, x10^3^/µl, median (IQR) | 8.3 (6.5-11.0) | 8.8 (6.6-11.6) | 8.6 (6.5-11.2) | 0.38 |
| Eosinophil count, x10^3^/µl, median (IQR) | 0.1 (0.1-0.3) | 0.2 (0.1-0.2) | 0.1 (0.1-0.3) | 0.13 |
| Haemoglobin, g/dl, mean (SD) | 13.7 (1.4) | 13.6 (1.3) | 13.6 (1.4) | 0.5 |
| Haematocrit level, %, mean (SD) | 37.8 (4.2) | 37.4 (3.9) | 37.6 (4.1) | 0.52 |
| C-reactive protein, mg/l, median (IQR) | 0.7 (0.4-2.0) | 0.7 (0.5-2.1) | 0.7 (0.5-2.0) | 0.52 |
| Interleukin 6, pg/ml, median (IQR) | 3.4 (2.0-7.4) | 2.6 (2.0-13.3) | 3.2 (2.0-8.1) | 0.77 |
| Ferritin, ng/ml, median (IQR) | 116.6 (52.6-176.4) | 67.8 (39.2-228.2) | 114.6 (51.7-180.7) | 0.44 |
| LDH, U/l, median (IQR) | 207 (177.0-236.0) | 200.0 (169.0-286.0) | 207.0 (175.8-246.3) | 0.52 |
| CPK, U/l, median (IQR) | 60.0 (41.0-96.3) | 66.0 (44.8-106.5) | 62.5 (41.0-98.3) | 0.58 |
| ALAT, U/l, median (IQR) | 12.6 (10.2-16.4) | 13.1 (10.1-17.4) | 12.8 (10.2-17.0) | 0.35 |
| Creatinine, µmol/l, mean (SD) | 52.6 (19.3) | 53.4 (18.6) | 53.0 (18.9) | 0.55 |
| Albumin, g/l, mean (SD) | 39.2 (4.4) | 46.0 (15.2) | 40.1 (7.3) | **0.002** |
| Acute pancreatitis (lipase >180 U/l, amylase >300 U/l), n (%) | 4 (3.5) | 3 (2.3) | 7 (2.9) | 0.71 |
| HDL, mmol/l, mean (SD) | 1.2 (0.3) | 1.2 (0.3) | 1.2 (0.3) | 0.23 |
| LDL, mmol/l, mean (SD) | 4.9 (2.1) | 2.8 (1.2) | 4.2 (1.8) | 0.27 |
| Triglycerides, mmol/l, mean (SD) | 1.3 (1.2) | 1.4 (0.7) | 1.3 (1.1) | 0.87 |
| Total cholesterol, mmol/l, mean (SD) | 4.1 (1.2) | 4.1 (1.2) | 4.1 (1.2) | 0.58 |
| Phosphorus, mmol/l, mean (SD) | 1.3 (0.5) | 1.3 (0.4) | 1.3 (0.4) | 0.15 |
| TSH, mU/l, median (IQR) | 2.2 (1.6-3.3) | 2.5 (1.7-3.4) | 2.4 (1.7-3.3) | 0.15 |
| Presence of anti-TPO antibodies, n (%) | 7 (38.9) | 11 (20.4) | 18 (25.0) | 0.13 |
| Presence of tissue transglutaminase IgA antibodies, n (%) | 9 (8.5) | 4 (6.7) | 13 (7.8) | 0.77 |

*Note.* IQR = interquartile range, SD = standard deviation, LDH = lactate dehydrogenase, CPK = creatine phosphokinase, ALAT = alanine aminotransferase, HDL = high density lipoprotein cholesterol, LDL = low density lipoprotein cholesterol, TSH = thyroid-stimulating hormone, anti-TPO = thyroid peroxidase antibodies

**
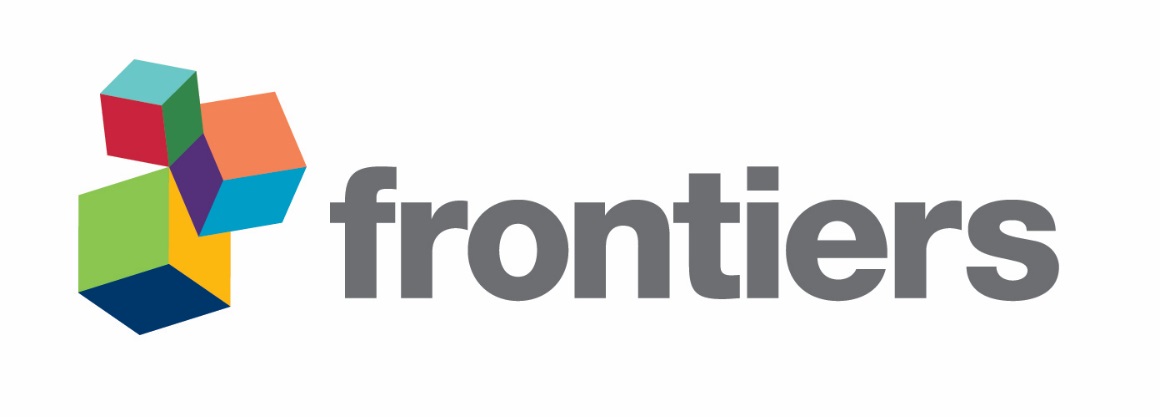
**
